# Using Instruction-Tuned Large Language Models to Identify Indicators of Vulnerability in Police Incident Narratives

**DOI:** 10.1007/s10940-025-09611-z

**Published:** 2025-06-17

**Authors:** Sam Relins, Daniel Birks, Charlie Lloyd

**Affiliations:** 1 School for Business and Society, University of York, York, UK; 2 School of Law, University of Leeds, Leeds, UK; 3 ESRC Vulnerability and Policing Futures Research Centre, York, UK

**Keywords:** Large Language Models, Unstructured Data, Policing, Vulnerability, Deductive Coding

## Abstract

**Objectives:**

Police routinely collect unstructured narrative reports of their interactions with civilians. These accounts have the potential to reveal the extent of police engagement with vulnerable populations. We test whether large language models (LLMs) can effectively replicate human qualitative coding of these narratives—a task that would otherwise be highly resource intensive.

**Methods:**

Using publicly available narrative reports from Boston Police Department, we compare human-generated and LLM-generated labels for four vulnerabilities: mental ill health, substance misuse, alcohol dependence, and homelessness. We assess multiple LLM sizes and prompting strategies, measure label variability through repeated prompts, and conduct counterfactual experiments to examine potential classification biases related to sex and race.

**Results:**

LLMs demonstrate high agreement with human coders in identifying narratives without vulnerabilities, particularly when repeated classifications are unanimous or near-unanimous. Human-LLM agreement improves with larger models and tailored prompting strategies, though effectiveness varies by vulnerability type. These findings suggest a human-LLM collaborative approach, where LLMs screen the majority of cases whilst humans review ambiguous instances, would significantly reduce manual coding requirements. Counterfactual analyses indicate minimal influence of subject sex and race on LLM classifications beyond those expected by chance.

**Conclusions:**

LLMs can substantially reduce resource requirements for analyzing large narrative datasets, whilst enhancing coding specificity and transparency, and enabling new approaches to replication and comparative analysis. These advances present promising opportunities for criminology and related fields.

## Introduction

The last decade has seen an increasing focus on the role of police in engaging with vulnerable populations. Police officers frequently come into contact with individuals experiencing mental health crises (Kane et al. 2018; Wood et al. 2021), homelessness (Herring 2019; Kouyoumdjian et al. 2019), substance dependency (Winkelman et al. 2018; Zhang et al. 2022) or exhibiting other complex needs. This shift has led to a reconceptualization of policing, moving away from the traditional ‘warrior’ model focused solely on crime control, toward a ‘guardian’ approach rooted in public protection, care, and community wellbeing (Engel 2015; Wood and Watson 2017; Koziarski et al. 2021). In response, public health approaches to policing recognize that many societal challenges, such as mental health issues and addiction, require interventions that reach beyond traditional mechanisms of law enforcement, and instead advocate for multi-agency working, upstream preventive measures, and community-based support rather than punitive responses (Christmas and Srivastava 2019; Van Dijk et al. 2019). Concurrently, trauma-informed practices in policing have also gained considerable traction in recent years, encouraging law enforcement to prioritize empathy, de-escalation, and compassionate communication, with the aim of reducing harm and enhancing community relations (Ko et al. 2008).

Despite its policy relevance, quantifying the extent of police engagement with vulnerable populations remains challenging. Current quantitative estimates often rely on ‘flags’ or ‘markers’ — categorical data fields recorded in call and dispatch systems to indicate the presence of specific incident characteristics, which can include the presence of one or more predetermined types of vulnerability. The validity of such data as a means to measure police contact with vulnerable populations is questionable for a number of reasons. First, categorical indicators struggle to adequately capture the complexity of situations police often find themselves in, where the intersection of multiple vulnerabilities may blur the boundaries of predefined categories. Second, the identification of a given vulnerability depends on the judgement and discretion of police officers or call handlers, who may differ significantly in their assessments of specific situations. Third, markers may be inconsistently applied across incidents, potentially varying across individuals recording data and across incident types, creating disparities in how vulnerability is recognized, flagged and recorded.

These challenges are likely reflected in the significant variation observed in efforts to quantify police engagement with vulnerability. To illustrate, relying on several representative snapshots of police demand, the UK's Policing Productivity Review (National Police Chiefs’ Council, 2023) estimated that between 5 and 9% of incidents involve mental ill health. Conversely, evidence submitted to a UK Parliamentary Inquiry into Policing and Mental Health on behalf of all UK Police Forces estimated that 20% of police time was spent dealing with mental health related calls (Home Affairs Select Committee 2015a), and that over 40% of calls for service were associated with those deemed vulnerable (Home Affairs Select Committee 2015b). Similarly, a systematic review of 15 studies conducted in North America estimated that approximately 1% of calls for service involved individuals with mental disorders (Livingston 2016). However, estimates varied substantially depending on the identification method used, ranging from 1% in dispatcher coding to 6% in police officer surveys and 9% in fieldworker observations. Such disparities between measurement approaches underscore the limitations of routine data collection, and the broader challenge of defining and recognizing vulnerability in policing contexts.

One potentially rich source of information in this context lies in the unstructured text narratives that police officers or call center staff record during routine logging of incidents or calls-for-service. These narratives typically document the circumstances, behaviors, and contextual details surrounding an incident and are collected for a variety of operational reasons including providing context for responding officers, documenting events for evidentiary purposes, and ensuring accountability and oversight. Yet despite their potential to provide insights beyond standardized data fields, such narratives remain largely underutilized in efforts aimed at quantifying police involvement with vulnerability. The primary reason for this lies in the resource-intensive nature of traditional analytical methods capable of deriving insights from unstructured data, which demand significant manual effort and are often infeasible at scale. Ultimately, this may limit agencies’ ability to access detailed insights that could otherwise support evidence-based problem and demand analyses, training, and inter-agency coordination (Dixon and Birks 2021).

Recent advances in large language models (LLMs) offer new ways to automate the processing of unstructured text data. The latest instruction-tuned LLMs (IT-LLMs) are designed to interpret and respond to natural language instructions directly, enabling them to flexibly support complex tasks like qualitative coding without additional specialized training (Zhang et al. 2024). By enabling scalable, qualitative coding of free-text data, IT-LLMs may provide viable means to bridge the gap between the limited scope of structured data and the detailed but labor-intensive nature of narrative analysis. In this study, we assess the capacity of IT-LLMs to replicate a deductive coding exercise: using unstructured incident narratives from Boston Police Department, we prompt LLMs to generate labels designed to identify situations associated with (i) mental ill health; (ii) substance misuse; (iii) alcohol dependence; and (iv) homelessness. We then compare the LLM-generated labels with those produced by non-expert human coders. Rather than seeking to definitively estimate the prevalence of vulnerabilities within this specific dataset, our primary aim is to explore the viability of a scalable methodology that could subsequently be deployed to generate such estimates.

### The Development of Instruction Tuned Large Language Models

Over the past decade, the capabilities of generative language models—artificial intelligence systems designed to produce human-like text—have transformed dramatically. At their core, these models are trained to perform a simple task: predicting the next word in a sequence based on the preceding words. Given the phrase “The sun is shining”, for example, a language model might suggest “brightly” or “today” as natural continuations, drawing on patterns of word usage in its training data. Early progress in applying machine learning to natural language tasks lagged behind other domains, such as computer vision. While Recurrent Neural Networks (RNNs) (Elman 1990) and their variant Long Short-Term Memory (LSTM) networks (Hochreiter and Schmidhuber 1997) marked important early breakthroughs in language modelling, they faced significant limitations. These models could generate coherent text in small fragments but struggled with longer passages (Bengio et al. 1994). Their sequential processing of inputs also made them computationally inefficient, limiting both model size and training capacity.

The introduction of the transformer architecture in 2017 marked a pivotal advancement in generative language models (Vaswani et al. 2017). Transformers overcame the long-range dependency issue present in earlier models through the use of a self-attention mechanism, which allowed them to effectively capture relationships between distant words in a sequence. More importantly, transformers could process text in parallel rather than sequentially, enabling far greater computational efficiency and allowing models to scale up significantly in both size and training data. Early transformer-based models demonstrated remarkable improvements in formal language representation compared to RNNs and LSTMs, producing long, coherent texts with correct grammar and syntax. As these models grew in scale, they began exhibiting ‘emergent capabilities’— performing a diverse range of tasks without any task-specific pre-training (Radford et al. 2019; Brown et al. 2020). These behaviors demonstrated the potential for language models to generalize across a wider range of applications than previously anticipated.

The current generation of instruction-tuned language models represents a further significant advance in this trajectory. These models are explicitly trained to interpret and follow natural language instructions, enabling them to adapt flexibly to diverse tasks while maintaining coherent reasoning (Mishra et al. 2022; Wei et al. 2022). Models such as GPT, Claude, and Llama can now engage in sophisticated tasks including analysis, summarization, and complex problem-solving—activities that previously required human expertise (Kojima et al. 2022; Srivastava et al. 2023). This capability to follow explicit instructions while drawing on broad knowledge has transformed these models from simple text generators into versatile tools for knowledge work, opening new possibilities for automating complex cognitive tasks that were previously considered beyond the reach of computational approaches.

### Related Work

The emergence of IT-LLMs offers new possibilities for criminological research, where text analysis has traditionally relied on conventional natural language processing (NLP) approaches. These established methods have made substantial contributions to policing research through rule-based systems and unsupervised methods for crime classification, entity extraction, and summarization (Ku et al 2008; Hughes et al 2008; Elzinga et al 2010; Poelmans et al 2011; Kuang et al 2017; Guetterman et al 2018; Karystianis et al 2018, 2019, 2024; Johnsen and Franke 2019; Birks et al 2020; Lwin Tun and Birks 2023). More recent applications of supervised learning and early transformer models like BERT have further advanced these capabilities (Haleem et al 2019; Osorio and Beltran 2020; Langton et al 2021; Halford et al 2022; Barros et al 2023; Hodgkinson et al 2023). However, these approaches typically require extensive pre-processing, careful parameter tuning, and task-specific training to achieve optimal results. This technical overhead can limit their adaptability to new classification tasks and make them challenging to integrate into existing research workflows without considerable customization.

Instruction-tuned large language models (IT-LLMs) represent a potentially transformative approach to these challenges. Unlike traditional NLP methods that rely on statistical patterns or pre-defined rules, IT-LLMs can interpret and apply complex classification criteria through natural language instructions. This capability offers several key advantages: first, they can process raw text without extensive pre-processing; second, they can adapt to new classification schemes without technical reconfiguration; and third, they are capable of providing natural language rationalizations for their decisions.

Capitalizing on these strengths, evidence for the potential of IT-LLMs in qualitative coding has emerged in a range of contexts. Studies examining their application to deductive coding tasks—where texts are systematically labeled according to predefined categories—have demonstrated their ability to replicate human-generated labels with high reliability (Xiao et al 2023; Chew et al 2023; Ashwin et al 2023; Tai et al 2024; Dunivin 2024). This research has explored various methodological considerations, including the impact of different prompting techniques (zero-shot, few-shot, and chain-of-thought reasoning) and model sizes on coding accuracy (Xiao et al 2023; Dunivin 2024). Critical examinations of output consistency (Tai et al 2024) and potential demographic biases (Ashwin et al 2023) have also highlighted important considerations for their deployment in research contexts.

Nevertheless, while several studies have discussed potential use cases of IT-LLMs in policing (Dubravova et al 2024; Adams 2024; Puczyńska et al 2024), this study provides the first empirical assessment of IT-LLMs'capacity for qualitative analysis in a policing context, specifically their potential to perform deductive coding to identify indicators of vulnerabilities in unstructured police narratives. We also investigate the effects of model size and prompting strategies on labelling accuracy and consistency across repeated prompts. Finally, given significant concerns regarding AI bias in criminal justice contexts, we conduct counterfactual analyses to assess potential biases in LLM outputs, systematically testing for demographic influences on coding tasks. Collectively, this approach provides valuable new insights into the application of IT-LLMs in policing research and methodological considerations for using LLMs in qualitative coding more broadly.

## Our Approach

### Dataset

We evaluated IT-LLMs'effectiveness in deductive coding using narrative data from the Boston Police Department's field interrogation and observation (FIO) dataset (Analyze Boston, n.d.). These narratives consist of free-text descriptions that document police interactions with the public, including sufficient contextual detail to identify vulnerabilities such as homelessness and substance abuse when present. The data are released under an Open Data Commons Public Domain Dedication and License (PDDL),^1^permitting both their use with commercial LLM services and enabling other researchers to independently replicate our analysis.

Two example narratives are included below—note the use of redaction to remove person-specific identifiers, and the use of domain specific shorthand:

Example 1:

“xxx has been seen walking on dorchester ave and hanging in fields corner. xxx spoke with officers and stated that she has a drinking problem and is homeless and hangs in the fields corner area. h983 sgt det cullity to be notified. very minor bop. hk01f—fritch/moccia”.

Example 2:

“officers observed xxx in the area, approaching multiple pedestrians, in the street, and on the sidewalk. xxx was observed constantly walking back and forth on the street, on dorchester ave. officers conducted a threshold inquiry, xxx stated he was looking for directions to jfk/red line, then recanted and said he was looking to meet a girl to possibly have drinks, and also said that he is in aa. he lives in hingham. distribution of class b on record. for intel fio—taylor/moccia h425f”.

The dataset was processed programmatically to prepare it for analysis. Narrative texts were extracted from all records where narratives were available, recorded between June 2015 and December 2023. The data was then cleaned by adding spaces after punctuation and redacted content (“XXX”), removing unnecessary whitespace, eliminating duplicate records, and excluding narratives with fewer than 200 characters.^2^ This process resulted in a final dataset comprising 32,218 unique narrative texts with a median word count of 81.

### Codebook Development

We developed a codebook focusing on four specific vulnerabilities: mental ill health, substance misuse, alcohol dependence, and homelessness. These vulnerabilities were selected on the basis that research has shown them to be frequently present among police interactions with suspects or in the course of police patrols (e.g. Johnsen and Fitzpatrick 2008; Greer et al. 2018; Robinson 2019; Wittmann et al. 2021) and because they are relatively easily defined and recognizable to non-experts. To develop the codebook, we manually selected 100 narratives from those used in prompt development (discussed in Sect. 2.3 below) detailing a range of cases relating to the selected vulnerabilities, from clear examples to those with indirect or circumstantial elements, providing a basis for determining the threshold of evidence required to identify each vulnerability. Each member of the research team independently coded these narratives based on their intuitive understanding. We then compared and discussed these initial codes to reach consensus definitions for each vulnerability which formed our codebook. The final definitions included detailed criteria and examples, designed such that non-expert audiences could apply them without needing further input.

Recognizing that many examples contained ambiguous evidence, we adopted a three-tiered labelling scheme: positive, inconclusive, and negative. This approach allowed us to better capture the uncertainty inherent in many narratives, where vulnerability indicators were often implicit rather than explicit (e.g., circumstantial cues rather than direct statements). By including an inconclusive category, we aimed to accommodate this ambiguity without forcing definitive positive or negative labels on cases lacking clear evidence.

Appendix A contains codebook definitions of all vulnerabilities considered.

### Prompt Development

The language models were provided with text instructions, known as prompts, that describe the deductive coding task. The design and choice of prompts are important, as they affect the model’s behavior and the quality of its outputs, as shown in numerous recent studies exploring the performance of IT-LLMs (White et al. 2023). In this study, we tested two prompting approaches: a basic codebook prompt that used the codebook definitions verbatim and a custom prompt iteratively refined to optimize the model’s performance. Note that the narrative data used in the development and testing of prompts were distinct from that used in all subsequent experiments.

#### Codebook Prompt

The codebook prompt quoted the definitions of vulnerabilities from the codebook verbatim. This approach was designed to directly compare the model’s ability to interpret the same information that would be provided to human coders, and to investigate whether definitions designed for humans are sufficient for language models to follow accurately. Additionally, this method closely mirrors the traditional manual coding process, differing primarily in substituting the human coder with an LLM.

The codebook prompt is a minimal template based on the codebook definitions. It instructs the model to read the codebook definition and then classify police narratives using that definition. The prompt instructs the model on the desired response: brief notes highlighting relevant quotes from the narrative and linking them to the respective parts of the codebook definition. This approach was informed by “chain of thought prompting” (Wei et al. 2022), where the language model is instructed to output intermediate reasoning steps before giving a final answer. Subsequently, the model is instructed to generate a classification in a pre-defined format that can be parsed by a processing script. If the model’s response fails to be parsed correctly, it is sent an additional message asking for reformatting. This process is repeated up to three times, after which, if the response is still incorrect it is marked as empty/missing.

To maintain a valid comparison between the LLM’s interpretation of codebook definitions and human coding, we deliberately kept the template instructions for the model simple and refrained from experimenting with them to improve classifications.

#### Custom Prompt

In contrast to the codebook prompt, we also developed custom prompts to optimize the instructions specifically for the LLMs. This approach, known as “prompt engineering”, involves iteratively testing and improving the instructions given to a language model to achieve the desired model behavior. Initially, the custom prompt began as a minimal set of instructions, asking the model to identify instances of a given vulnerability, without providing a specific definition of that vulnerability, and to label them as positive, inconclusive, or negative based on the evidence present in the narrative. This approach relies on the model’s ‘understanding’ of concepts such as mental ill health as encoded through its training data. This baseline prompt was then refined by analyzing the LLM’s outputs and adding or rephrasing instructions to address any errors or biases observed in its responses. This process allowed us to fine-tune the instructions to better suit the LLM’s strengths and limitations and reduce the size and complexity of instructions with comparison to the codebook prompts, which can be advantageous especially for smaller models.

The final custom prompt template reflects this process of iterative refinement. The template retains the core task description and labelling scheme from the initial minimal prompt but includes more detailed and specific instructions. We added phrases like “contains unmistakable evidence of, having ruled out any other plausible explanations” and “evidence that is best explained by… but there is not definitive or conclusive confirmation…” based on initial experiments showing the model was too permissive in its assignment of positive and inconclusive labels. Additional vulnerability-specific instructions were added to the template to address any specific mistakes and steer toward the desired classifications for each vulnerability.

Appendix B contains details of both codebook and custom prompts used in our experiments.

### LLMs

In addition to testing different prompting strategies, we evaluated different IT-LLMs to assess their capabilities for coding tasks. Recent advancements have produced a range of models differing in size, measured by the number of parameters, and whether they are open-source or proprietary. These factors significantly influence performance, cost, and suitability for deployment in circumstances where computational resources are scarce or where data security considerations limit the sharing of data with third parties.

LLMs range in size from small models with around 1 billion parameters to extremely large models exceeding 500 billion parameters. Larger models tend to excel in handling complex and verbose instructions, performing better in tasks requiring nuanced understanding and reasoning skills. However, this comes at the cost of requiring advanced and expensive hardware, typically accessed through cloud-computing services. Smaller models, though generally less capable in handling complex tasks, are far more computationally efficient. They can run on consumer-grade hardware, such as laptops or smartphones, making them both cost-effective and widely accessible. This efficiency makes them ideal for scenarios with limited computational resources or strict data security requirements that preclude the use of cloud-based services. Balancing these trade-offs—between performance, cost, and deployment constraints—is critical when selecting a model, especially when working with sensitive and potentially identifiable data, such as police narratives, that are likely to have strict information governance requirements.^3^

Another important distinction is between open-source and proprietary models. Open-source models, freely available with permissive licenses, allow researchers to preserve specific model versions and share them alongside their methodology, ensuring replicability. They can also be deployed on private infrastructure, facilitating work with sensitive data. By contrast, proprietary models from companies like OpenAI and Google, though often superior in performance, are accessible only through cloud APIs creating dependencies on external infrastructure and potential changes in architecture, pricing, or access that may hinder replication.

For our study, we tested models of varying sizes and both open-source and proprietary nature to evaluate their performance. Specifically, we used the following models:

1. **Llama 8B and 70B**: These open-source models, released by Meta, have shown competitive performance relative to their size. The 8 billion parameter model represents a smaller, more accessible option, while the 70 billion parameter model provides a mid-sized alternative with enhanced capabilities.
2. **GPT-4o**: This proprietary model from OpenAI is rumored to have over 1 trillion parameters, representing the state-of-the-art in LLM performance at the time of writing. While the exact size is undisclosed, GPT-4o is known for its advanced capabilities and state-of-the-art performance across a wide range of tasks.

Hereon, we shall use the term *"labelling-configuration"* to describe each unique combination of a model and a prompting strategy used to classify narratives. For example, the configuration “Codebook 70B” refers to using the codebook prompt with the Llama 70B model.

### Label Variability

IT-LLMs generate text probabilistically, meaning they may produce different outputs given identical inputs. For our coding task, this means a single narrative could receive different vulnerability labels across multiple classifications, even when using the same model and prompt. To assess this potential variability in coding decisions, we classified each narrative ten times for each vulnerability using identical labelling configurations and analyzed the consistency of these repeated classifications.

### Evaluation Dataset

We began by using Llama-based models (7B and 80B variants with both custom and codebook prompts) to code 4,000 randomly selected narratives.^4^ This approach helped estimate the level of class-imbalance within our dataset—recognizing that certain vulnerabilities were unlikely to be prevalent across all narratives—and in turn directed a purposive sampling of a subset of narratives for evaluation. For each narrative, we generated ten labels per labelling configuration to capture the inherent variability in LLM outputs. We then implemented a consensus approach: each narrative's final label was determined by the majority across its ten generated labels. Where no majority emerged, the narrative was marked as inconclusive.

Analysis of these labels (see Appendix C) indicates that the majority of police narratives were classified as not containing evidence of the specified vulnerabilities. Custom prompt configurations consistently produce high proportions of negative classifications, typically above 90% of narratives, whilst codebook prompts demonstrate greater variability in their classifications, particularly when used with smaller models. This pattern is further emphasized when examining unanimous classifications (where all ten iterations produced the same label): custom prompts achieve markedly higher rates of unanimous negative classifications compared to codebook prompts, with this effect most pronounced when comparing smaller models.

Informed by these analyses, we designed a sampling method to include a higher proportion of positive and inconclusive labels for each vulnerability when selecting an evaluation subset of 500 narratives to be coded by humans.^5^ To ensure a balanced evaluation, we used the consensus labels from the custom 70B configuration as our reference, based on our preliminary experimentation, that suggested larger models with custom prompts provide the most accurate labels. We randomly selected 100 examples labelled as negative for each of the four vulnerabilities. We then randomly selected 50 examples labelled as positive and 50 labelled as inconclusive for each of the four vulnerabilities—given that several examples had non-negative labels for multiple vulnerabilities, the final label proportions were actually greater than 50. For alcohol dependence, however, only 15 examples were marked positive, so we supplemented this with 35 inconclusive examples to maintain sample size.

Due to the high API costs associated with using GPT-4o, we limited our coding of the police narratives to the evaluation dataset of 500 narratives, as opposed to the 4000 narratives coded by the Llama configurations. We also chose to limit the evaluation of GPT-4o to the codebook prompt only, without developing a custom prompt. The decision to avoid a custom prompt for GPT-4o was based on the substantial API costs of prompt refinement, and that our initial experiments didn’t suggest that there would be much value in testing a custom prompt: we proposed that the GPT-4o model (the largest utilized) would be best suited to the detailed and lengthy instructions in the codebook, and that custom prompts would be most valuable to smaller models, where more carefully worded instructions can yield more dramatic improvements in outputs.

To provide appropriate comparator labels for the 500 examples, we recruited two coders who had not previously conducted qualitative coding of incident narratives and were not professionally involved in vulnerability research or service provision. We selected coders without prior domain experience to evaluate the baseline effectiveness of the codebook definitions free from pre-existing knowledge that might lead to deviation from the specified definitions. Each coder received the codebook definitions for each vulnerability and basic instructions to code each narrative for the four vulnerabilities. They worked independently without conferring and were instructed to use their own intuition whenever the guidance in the codebook was unclear. The human coders'results were subsequently reviewed by the research team, who adjudicated disagreements to reach a consensus and ensure a single set of human labels for comparison with the LLM outputs. The numbers (and percentages) of examples requiring adjudication were as follows: mental ill health 49 (9.8%); substance misuse 118 (23.6%); alcohol dependence 55 (11%); homelessness 50 (10%).

### Reproducibility Materials

To facilitate replication and promote transparency, we have made all data preprocessing, label generation and analysis scripts publicly available. The complete dataset used in this analysis is archived at https://osf.io/har9m/, while all analysis scripts and code are available in a version-controlled repository at https://github.com/samrelins/vulnerability_classifier_pipeline. These materials allow for complete reproduction of all results and figures presented in this paper.

## Analysis & Results

### LLM Consensus vs Humans

The following analyses compare LLM consensus labels from each labelling-configuration with those generated by human coders. As discussed previously, the LLM consensus label was determined by majority vote among ten labels generated for each narrative. If no majority label was present, the label was marked as inconclusive.

#### Error Analysis

To quantify disagreement between the LLM consensus and the human labels, we assigned numerical values to each label: negative (0), inconclusive (1), and positive (2). We then calculated the mean squared error (MSE) for each set of LLM labels compared to the human labels. The MSE is given by Eq. (1):

1 $$MSE=\frac{1}{n}{\sum }_{i=1}^{n}{\left({y}_{i}-{\widehat{y}}_{i}\right)}^{2}$$

where ${y}_{i}$ represents the human label for example $i$, ${\widehat{y}}_{i}$ represents the LLM consensus for the same example, and $n$ is the number of examples. Scoring labels as 0, 1, and 2 allows MSE to capture the severity of disagreements, penalising larger mismatches (e.g., negative vs positive) more than smaller ones (e.g., negative vs inconclusive).

**Fig. 1:**
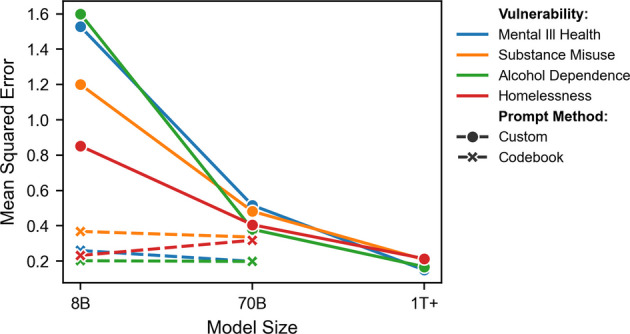
Mean squared error (MSE) between human and LLM consensus labels across different model sizes (8B, 70B, and 1T +) and prompt methods (Custom and Codebook) for four vulnerability types. Solid lines with circles represent Custom prompts, while dashed lines with crosses represent Codebook prompts

Figure 1 displays the MSE results for each LLM size and prompting method. A clear trend is observed in the codebook configurations, with errors decreasing as model size increases. Errors decrease significantly from the 8B model to the 70B model, with a smaller decline for the larger GPT-4o model. This trend demonstrates a general improvement in alignment with human labels for the codebook prompting approach as model size increases. The custom prompt configurations exhibit considerably lower errors compared to their codebook counterparts and are comparable in performance to the much larger GPT-4o codebook configuration. However, unlike the codebook configurations, there is no obvious trend in MSE reduction as model size increases for the custom configurations. The errors fall slightly for mental ill health, remain largely the same for substance misuse and alcohol dependence, and show a moderate increase for homelessness.

#### Precision, Recall, and F1 Score

To gain a more precise understanding of the performance of various labelling configurations, with particular focus on the less frequent positive and inconclusive labels, we calculated precision, recall and F1 scores. To do so, we grouped the inconclusive and positive labels into a combined “positive” category, treating the negative labels as a separate category. We then calculated the statistics as follows:

- **Precision:** Precision measures the accuracy of the positive labels assigned by the models. It is defined as the proportion of true positive labels among the positive labels the model assigned, shown in Eq. (2):
  2 $$Precision=\frac{True Positives}{True Positives+False Positives}$$
  A higher precision indicates that the model makes fewer false positive errors, meaning that the positive identifications are more likely to be correct.
- **Recall:** Recall, also known as sensitivity, assesses the model’s ability to identify all actual positive cases. It is defined in Eq. (3), as the proportion of true positive cases among all actual positive cases:
  3 $$Recall=\frac{True Positives}{True Positives+False Negatives}$$
  A higher recall indicates that the model is more effective at detecting positive cases, reducing the number of false negatives.
- **F1 Score:** The F1 score provides a balance between precision and recall, offering a single metric that accounts for both false positives and false negatives, shown in Eq. (4):
  4 $$F1 Score=2\times \frac{Precision\times Recall}{Precision+Recall}$$
  F1 is particularly useful when dealing with imbalanced datasets, as it harmonizes the need for both high precision and high recall. A higher F1 score indicates a better overall performance of the model in classifying the positive labels correctly while minimizing both types of errors.

**Fig. 2:**
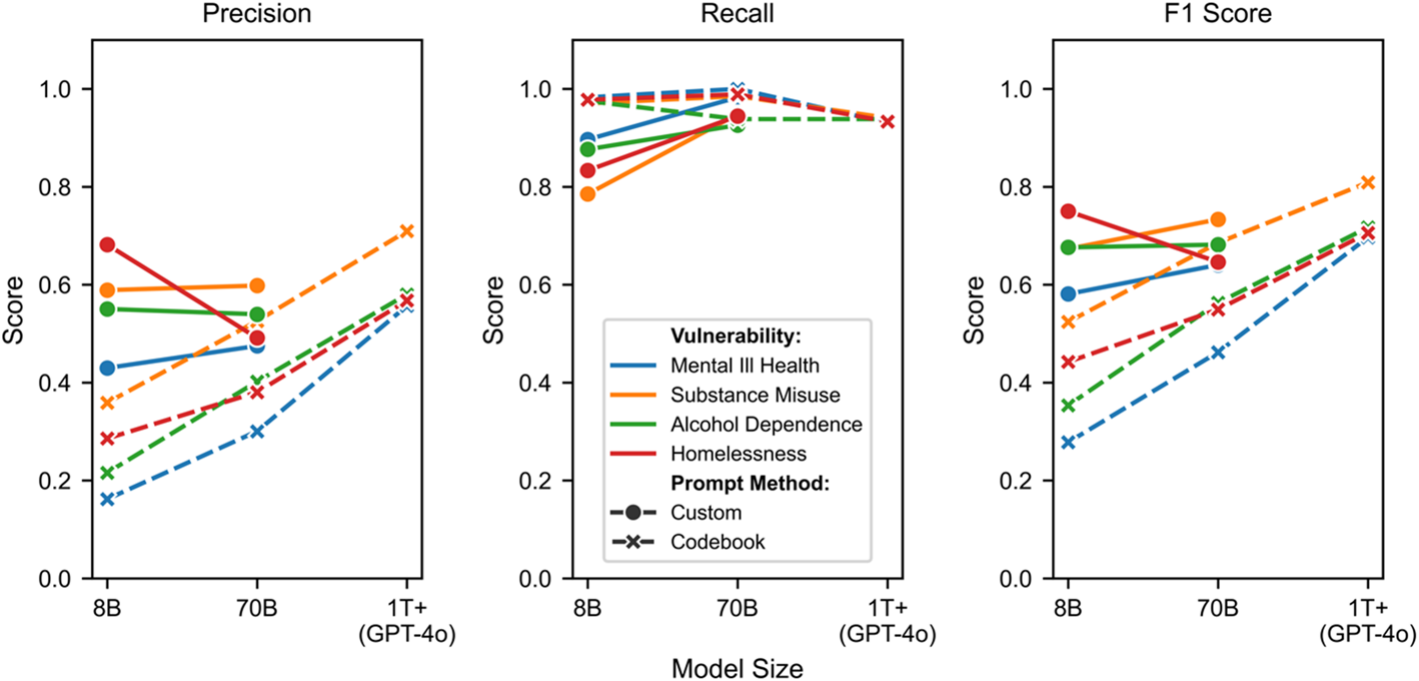
Precision, recall, and F1 scores for positive + inconclusive labels across different model sizes (8B, 70B, and 1 T +) and prompt methods (Custom and Codebook). Solid lines with circles represent Custom prompts, while dashed lines with crosses represent Codebook prompts

Figure 2 illustrates the precision, recall, and F1 scores for the combined positive labels across each labelling configuration. The results align with trends observed in the MSE analyses. Codebook configurations exhibit a clear upward trend in performance as model size increases, primarily driven by improved precision. This is evident in the consistent increase in F1 scores across all vulnerabilities, with mental ill health and alcohol dependence showing particularly marked improvements from below 0.4 for 8B models to above 0.6 for 1 T + models. Custom prompt configurations demonstrate substantially enhanced performance compared to their respective codebook variants, especially for smaller models. The 8B custom prompt models achieve F1 scores (approximately 0.6–0.7) comparable to those of 1 T + codebook prompt models for most vulnerabilities.

All configurations demonstrate consistently high recall statistics, clustering above 0.8 and frequently exceeding 0.9. While the overall performance of custom configurations shows no clear relationship with model size, a closer examination reveals a consistent upward trend in recall as models get larger—the corresponding precision scores vary considerably between vulnerabilities, leading to fluctuating F1 scores that partially mask this recall improvement. However, in contexts where positive examples are relatively rare, high recall may be more valuable than balanced performance. These results suggest a promising practical application: the models could serve as effective screening tools, reliably identifying negative examples that can be excluded from manual review. This would allow human coders to focus their limited resources on examining only those cases flagged as positive or inconclusive by the model, potentially offering significant efficiency gains even if precision remains imperfect.

#### Confusion Matrices

**Fig. 3:**
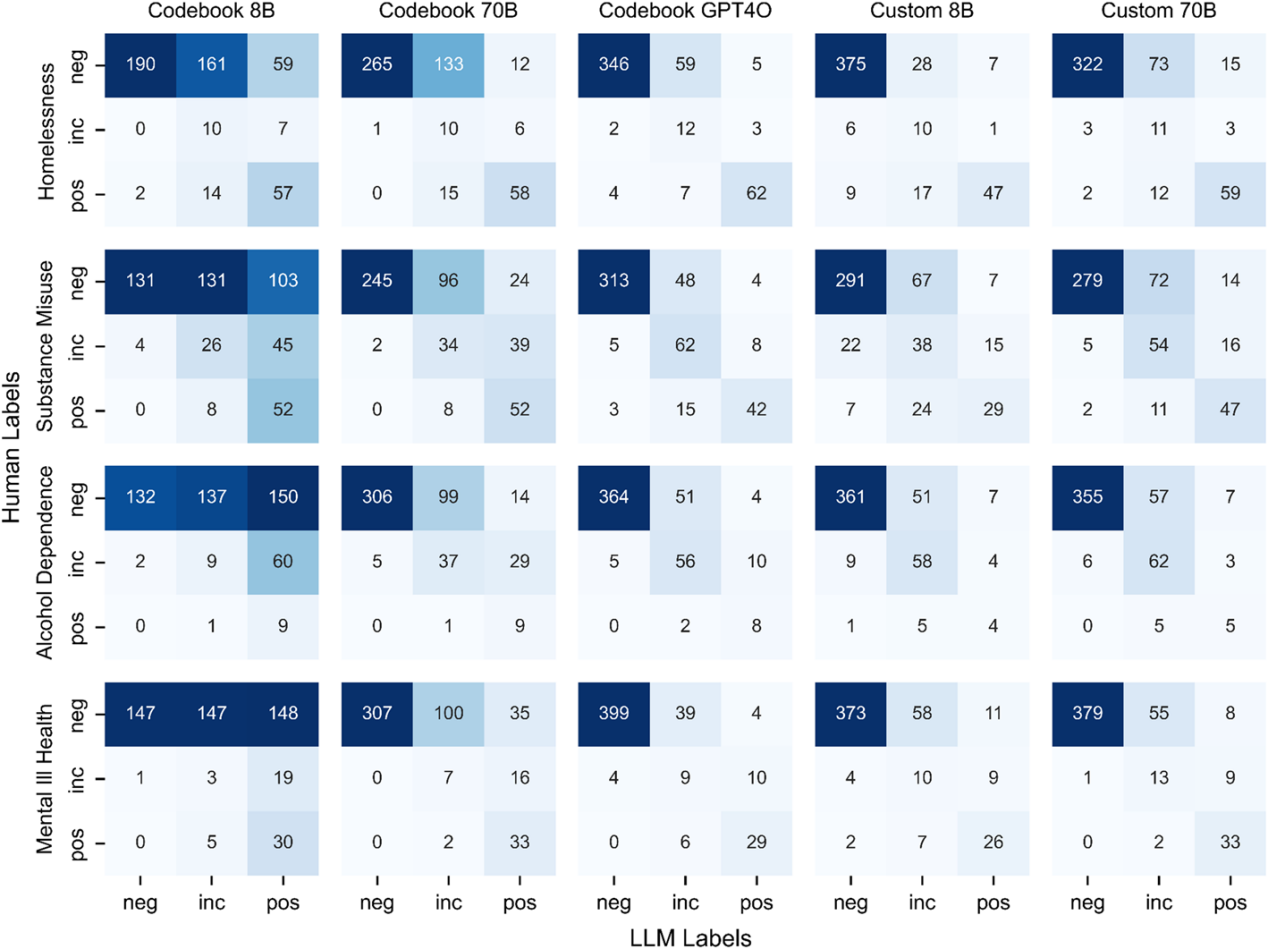
Confusion matrices comparing human labels (rows) with LLM consensus labels (columns) across different labelling configurations and vulnerability types. Cell values and shading intensity indicate the number of examples assigned each label combination. Darker shading indicates higher frequencies, with diagonal elements representing agreement between human and LLM labels. Results show strong alignment on negative classifications across all configurations, with most disagreements occurring at the boundaries between negative-inconclusive and inconclusive-positive categorizations

To further visualize the alignment between human and LLM labels, Fig. 3 shows confusion matrices for each labelling-configuration compared to the human labels. In each matrix, rows represent the human labels, columns represent the LLM labels, with the numbers in each cell (and the color of that cell) representing the number of examples assigned the respective labels. Within each square of nine cells for each labelling configuration, the cells along the diagonal from top-left to bottom-right represent agreement between the LLM and human labels, the off diagonals represent disagreement.

The confusion matrix analysis reveals additional nuances in model performance beyond those identified in the precision, recall, and F1 score metrics. Firstly, while all configurations demonstrate strong alignment with human coders on negative classifications, the analysis shows that disagreements primarily result from the LLMs over-assigning inconclusive labels to cases human coders judged as negative. This trend is particularly evident in the GPT-4o model, which generally aligns well with human assessments aside from a tendency to classify some human-negative cases as inconclusive. To illustrate, in Substance Misuse, 48 cases categorized by humans as negative were categorized by GPT-4o as inconclusive. A secondary pattern emerges with the custom prompt configurations where, unlike GPT-4o, they show more variability at the inconclusive-positive boundary, assigning positive labels where human coders were more conservative with an inconclusive classification or vice versa. This is most prominent with the 70B model, suggesting that while the larger model demonstrates a greater precision in negative classifications, it also introduces a greater degree of variability in cases deemed inconclusive or positive.

We have already discussed how the strong alignment with negative human labels suggest LLMs may be effective as initial filters for excluding clearly negative cases. However, these results also suggest there might be further strategies for reducing manual labelling requirements by focusing review efforts where model-human disagreements most frequently arise. For instance, with GPT-4o, focusing manual inspection solely on inconclusive labels could yield high overall accuracy, as the remaining classifications tend to align closely with human labels. A similar approach could be applied to the custom prompt configurations, although reviewing both inconclusive and positive cases may be advisable to refine estimates further, particularly where these models introduce variability between positive and inconclusive judgments.

### Label Variability

The consensus labels analyzed thus far represent only a summary of each model’s output. However, every example underwent 10 labelling iterations per vulnerability and labelling configuration. The following analyses explore the consistency and variability within these multiple label assignments.

#### Label Entropy

To quantify the consistency of model outputs, we calculated the entropy of labels across repeated classifications. Entropy, in this context, measures the uncertainty in the labels assigned by each model by analyzing variability in the ten classifications (positive, inconclusive, negative) generated for each example by a given labelling configuration. A higher entropy value indicates greater uncertainty or disagreement among the labels, while a lower entropy value suggests more consistent labelling. The entropy for a given narrative is calculated using the Shannon entropy formula in Eq. (5):

5 $$H=-{\sum }_{i=1}^{n}{p}_{i}{log}_{2}\left({p}_{i}\right)$$

where ${p}_{i}$ is the probability of label ($i$), and ($n$) is the number of possible labels (in this case, 3).

To estimate the average entropy and associated uncertainty for each labelling configuration we employed a bootstrapping approach. We resampled the entropy values with replacement 10,000 times, calculating the mean for each resample, and deriving the overall mean and 95% confidence intervals from the distribution of these resampled means.

**Fig. 4:**
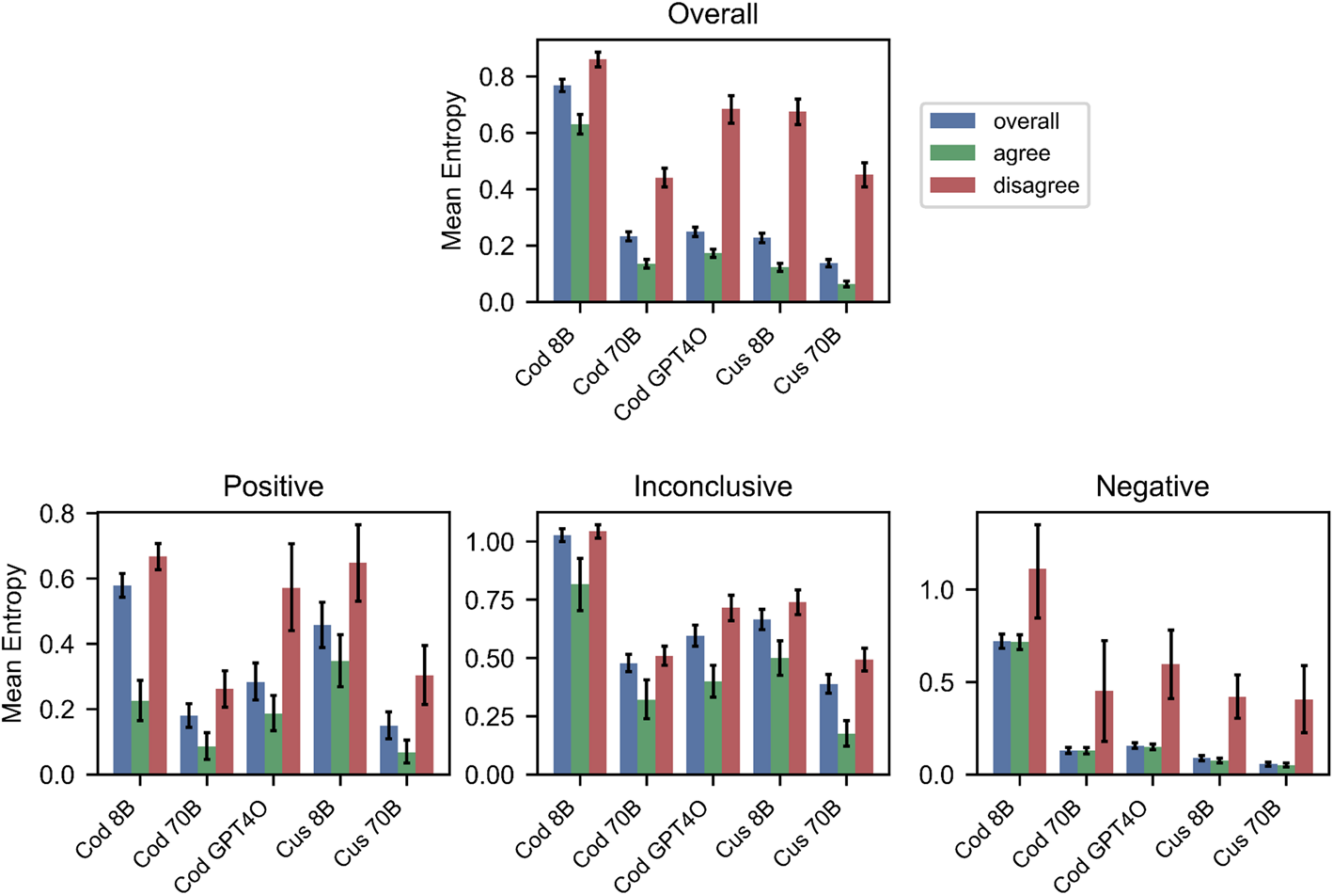
Mean entropy of LLM labels across different labelling configurations, with 95% confidence intervals derived from bootstrap resampling. The top panel shows overall entropy, while bottom panels show entropy stratified by consensus label type (Positive, Inconclusive, Negative). Within each panel, bars represent overall entropy (blue), entropy for examples where LLM and human labels agree (green), and entropy for examples where they disagree (red). Lower entropy values indicate more consistent labelling across repeated classifications, with clear patterns showing lower entropy when LLM and human labels agree

The entropy analysis in Fig. 4 reveals a consistent theme across different labelling configurations and outcome scenarios: as models assign labels that agree with human judgements, they also exhibit greater certainty in these decisions. This pattern manifests in several key observations.

Firstly, instances where the model consensus agrees with human labels consistently show lower entropy than instances of disagreement (demonstrated by the relative size of the green bars with respect to the red bars). This trend persists across all labeling configurations, and holds true when examining individual label categories (positive, negative, and inconclusive). This relationship between label entropy and human-model disagreement indicates that model uncertainty could be a useful tool for automatically identifying and prioritizing ambiguous cases for expert review, potentially improving overall labelling accuracy.

The relationship between model-human agreement and certainty is further reinforced by the trends observed across different labeling configurations. Consistent with earlier analyses comparing model outputs to human labels, labels from larger models and custom prompts generally exhibit lower entropy overall (though, a notable exception to this trend is the small increase in entropy between the 70B parameter model and GPT-4o). Moreover, the differences in the entropy values between examples agreeing with and disagreeing with human labels increases with model size, and when moving from the codebook to custom prompts, in line with the trends already observed. These findings indicate that as models become more adept at producing labels that align with human judgements, they also become more consistent in their classifications.

The label-specific entropy trends provide further evidence of this theme. The trends observed in the overall entropy statistics persist when divided into the different labels. Negative labels, which previous results showed to have the highest agreement between models and human coders, exhibit the lowest entropy overall. Conversely, inconclusive labels show the highest entropy overall, reflecting an inherent uncertainty in cases that, by definition, cannot be definitively classified. It is important to note that this higher entropy for inconclusive labels is partly deterministic, as our consensus method assigns an inconclusive label by default when there is no clear majority.

#### Model Consensus and Human Alignment

Previous analyses revealed that higher agreement among LLM-generated labels correlates with improved alignment to human labels. This suggests that model agreement could serve as a useful proxy for confidence in LLM classifications. To explore this further, we investigated the relationship between model consensus (the extent to which repeated classifications for the same narrative agree) and alignment with human labels. Additionally, we examined how much of the dataset achieves varying levels of agreement, providing insights into the potential of using consensus as a guide for selective human review.

For each narrative, we counted how frequently each label (positive, inconclusive, or negative) appeared across its ten classifications. We focused our analysis on cases where a single label was assigned 6 or more times, as this represents a clear majority that cannot be matched by the other labels combined. For example, if a model classified a narrative as ‘negative’ in 7 out of 10 iterations, this would represent an agreement level of 7.

For each agreement level, we assessed two key aspects:

1. **Proportion of Data by Agreement Level**: The percentage of narratives achieving each agreement level, stratified by label (positive, inconclusive, negative), vulnerability and labelling configuration. This quantifies how much of the dataset falls into categories with stronger or weaker model consensus.
2. **Alignment with Human Labels**: The proportion of classifications at each agreement level that matched human labels. This provides insights into how increasing agreement influences alignment, highlighting whether higher model consensus consistently leads to more accurate classifications.

**Fig. 5:**
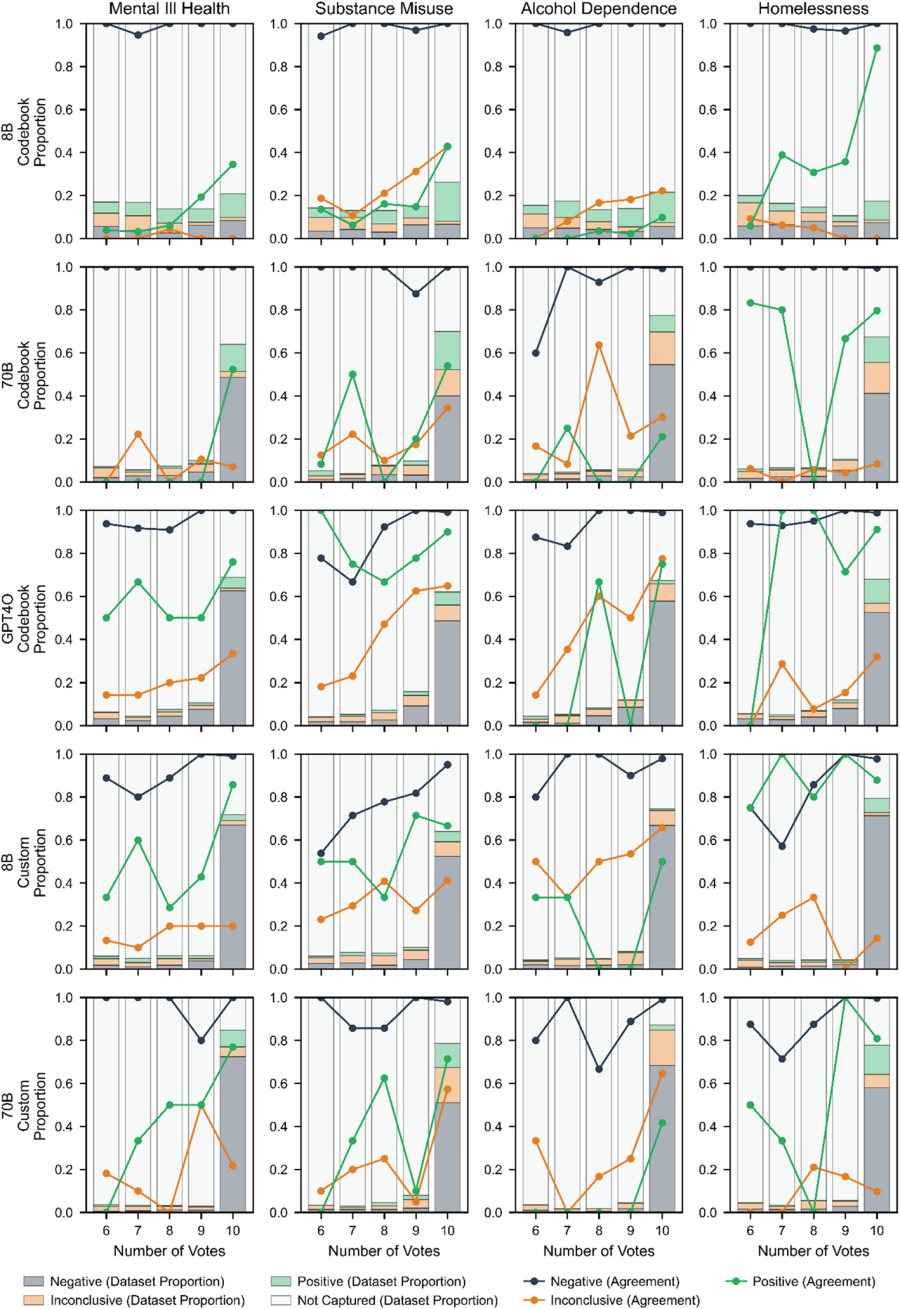
Relationship between model consensus and alignment with human labels across different labelling configurations and vulnerabilities. Stacked bars show the proportion of examples receiving 6–10 matching votes (x-axis) for each label type, with grey representing negative labels, orange representing inconclusive labels, and green representing positive labels. Line plots show the alignment between LLM and human labels at each consensus level for negative (black), inconclusive (orange), and positive (green) classifications. Higher consensus levels generally correspond to better alignment with human labels, particularly for negative classifications

We visualized the results using stacked bar plots to display the proportion of narratives at each agreement level and label, with overlaid line graphs showing alignment with human labels, in Fig. 5.

Mirroring our entropy analyses, there is a strong relationship between the degree of consensus among model-generated labels and their alignment with the human labels, as shown by the rising trend in the line plots across all configurations—the more votes that agree on the same label, the more those classifications tend to align with human labels. With the exception of the 8B Codebook configuration, there is also a clear trend for the models to assign a majority of examples a unanimous 10 votes, signified by the dominating bars rightmost of each subplot. These patterns are particularly evident in negative classifications, which constitute the majority of high-consensus cases among the three best-performing configurations (GPT-4o, Custom 70B, and Custom 8B), ranging from 48–72% of examples depending on the vulnerability and configuration, and aligning with human labels in greater than 95% of cases at the 10/10 agreement level. For instance, GPT-4o's unanimous negative classifications align with human labels at rates of 100% for mental ill health, 99.2% for substance misuse, 99% for alcohol dependence, and 98.9% for homelessness at the highest agreement level. These results extend our previous findings on the effectiveness of LLMs as negative filters, demonstrating that near-perfect alignment with human coding can be achieved by flagging less confident classifications for review, while still maintaining automated classification for the majority of negative cases.

The results also indicate that the alignment of positive classifications might be improved by selecting examples at higher agreement levels, though with more variation across vulnerabilities and labelling configurations. As shown by the green sections of the bars and corresponding green lines, GPT-4o achieves the highest proportion and alignment for positive classifications at maximum agreement, though these represent a relatively small portion of the total examples overall. For example, at agreement level 10, GPT-4o identifies 11.2% of homelessness cases as positive with 91% alignment, and 6% of substance misuse cases as positive with 90% alignment. However, performance varies significantly by vulnerability and labelling configuration: substance misuse and alcohol dependence positive labels are typically not as well aligned as the other vulnerabilities (though, in the case of alcohol dependence this is largely related to the lower number of positive examples in the sample). The variable performance across different vulnerabilities suggests that while agreement levels could be used to improve the reliability of positive classifications, the appropriate threshold for automated versus manual coding would need to be carefully calibrated based on both the specific concept being coded and one's tolerance for potential misclassification.

Inconclusive classifications show distinct patterns from positive and negative labels in how they distribute across agreement levels. Unlike negative and positive classifications, which tend to concentrate at higher agreement levels (especially 9–10 votes), inconclusive labels show a notably flatter distribution across agreement levels. This pattern is consistent across all model sizes and prompting strategies and aligns with our conceptual understanding of what"inconclusive"represents—cases where evidence is mixed or ambiguous rather than clearly indicating presence or absence. These results suggest that LLMs are replicating this ambiguity in their repeated classifications, effectively"disagreeing with themselves"about whether cases are truly inconclusive or better classified as positive or negative. This is particularly evident in the alignment curves, where inconclusive classifications consistently show lower alignment with human labels even at high agreement levels. For instance, while GPT-4o's negative classifications reach near-perfect alignment at agreement level 10, its inconclusive classifications rarely exceed 70–80% alignment even with maximum consensus. This pattern suggests that while LLMs can often effectively identify clear positive and negative cases, inconclusive classifications appear to warrant human review regardless of agreement level, reflecting the inherent complexity of cases where evidence is ambiguous or conflicting.

### Qualitative Insights

Both of our prompting approaches instructed the models to provide chain-of-thought (CoT) explanations alongside their classifications, with the primary aim of anchoring the outputs to specific content from the narratives and the prompt instructions, thereby reducing the likelihood of fabrications. While these explanations might not necessarily reveal the true reasoning behind the models’ classifications, they provide a practical tool for interpreting outputs by linking features of the input narrative to the given instructions and the final label. This can help identify where models may diverge from instructions or where their outputs appear inconsistent with human labels. In this context, the explanations offered a means of exploring potential patterns in model behavior, particularly in cases of disagreement.

With this in mind, we conducted a short qualitative analysis of cases where the custom 8B parameter configuration’s classifications for substance misuse disagreed with human labels. This particular labelling configuration and vulnerability were selected for review because, among the higher-performing models with potential practical applications, it demonstrated the greatest divergence from human labels, offering a greater number and potential variety of disagreements to explore. Analyses revealed patterns of misclassification, such as a tendency to misattribute “unusual” behaviors— nervousness or erratic actions—as evidence of substance misuse, even in the absence of clear associations or instructions emphasizing these behaviors. Additionally, the model sometimes demonstrated inconsistent adherence to specific instructions; for instance, while prompts explicitly excluded alcohol-related evidence as an indicator of substance misuse, the model would sometimes follow this instruction, but on other occasions would cite alcohol consumption as justification for a positive or inconclusive label. At the same time, the model consistently followed other more complex, nuanced instructions, indicating that its errors were not the result of a general inability to align with detailed or complicated instructions. Unfortunately, the limited scope of our analyses prevents us from generalizing these findings or identifying their underlying causes. Addressing these issues in depth would require a more systematic approach, with additional experimental data exploring model behavior across configurations, a greater number of human labelers and written explanations for each of the human labels.

### Counterfactual Analyses

To investigate potential biases in the language models and our methodology, we developed a counterfactual approach to assess the impact of key demographic characteristics on vulnerability classifications. This approach explores these potential biases by comparing model classifications for narratives where the sex and race descriptors of individuals are systematically manipulated.

To generate data for these analyses we selected a subset of 100 narratives from our original dataset, ensuring approximately equal representation of the four vulnerabilities and the presence of a single, clearly identifiable subject. These narratives were manually annotated with the race and sex of the subject as described in the original text. Using a custom script employing GPT-4o, we generated counterfactual versions of each narrative, systematically altering the race and sex descriptors across a predefined set of demographics (sex: unknown, female, male; race: unknown, Black, White, Hispanic, Asian). This process yielded a set of 1500 counterfactual narratives (100 narratives, with 15 different combinations of sex and race) identical in content to the originals, differing only in the demographic descriptors of the subject.

We then applied our original classification methodology to this new dataset of counterfactual narratives, using all labeling configurations to classify each narrative for the four vulnerabilities of interest. The resulting dataset allows us to examine the potential impact of race and sex on the models’ vulnerability classifications, thereby assessing any systematic biases in our approach.

We employed generalized linear mixed-effects models (GLMMs) to assess the impact of demographic characteristics on vulnerability classifications. Analyses were conducted using R (R Core Team 2024, version 4.3.2) with the lme4 package (Bates et al. 2015, version 1.1.35.5). For each combination of vulnerability and labeling configuration we fitted a GLMM with a binomial distribution and logit link function. The dependent variable was a binary outcome combining positive and inconclusive classifications (1) versus negative classifications (0). The model was specified as shown in Eq. (6):

6 $$log\left(\frac{{p}_{ij}}{1-{p}_{ij}}\right)={\beta }_{0}+{\beta }_{1}{Race}_{ij}+{\beta }_{2}{Sex}_{ij}+{u}_{j}$$

where ${p}_{ij}$ is the probability of a positive/inconclusive classification for observation $i$ in narrative $j$, ${\beta }_{0}$ is the intercept, ${\beta }_{1}$ and ${\beta }_{2}$ are the fixed effects for race and sex respectively (with ‘unknown’ as the reference category), and ${u}_{j}$ is the random intercept for each base narrative.

We calculated average marginal effects (AMEs) for each demographic characteristic using the margins package (Leeper 2024). AMEs represent the average change in the probability of a positive/inconclusive classification associated with each demographic category, relative to the ‘unknown’ reference category. For each marginal effect, we computed point estimates, standard errors, 95% confidence intervals, z-values, and p-values.

To address multiple comparisons, we applied the Holm-Bonferroni method to adjust p-values across all models and demographic characteristics tested. Effects with adjusted p-values < 0.05 were considered statistically significant, indicating a reliable association between the demographic characteristic and the probability of a positive/inconclusive vulnerability classification.

#### Counterfactual Results:

**Fig. 6:**
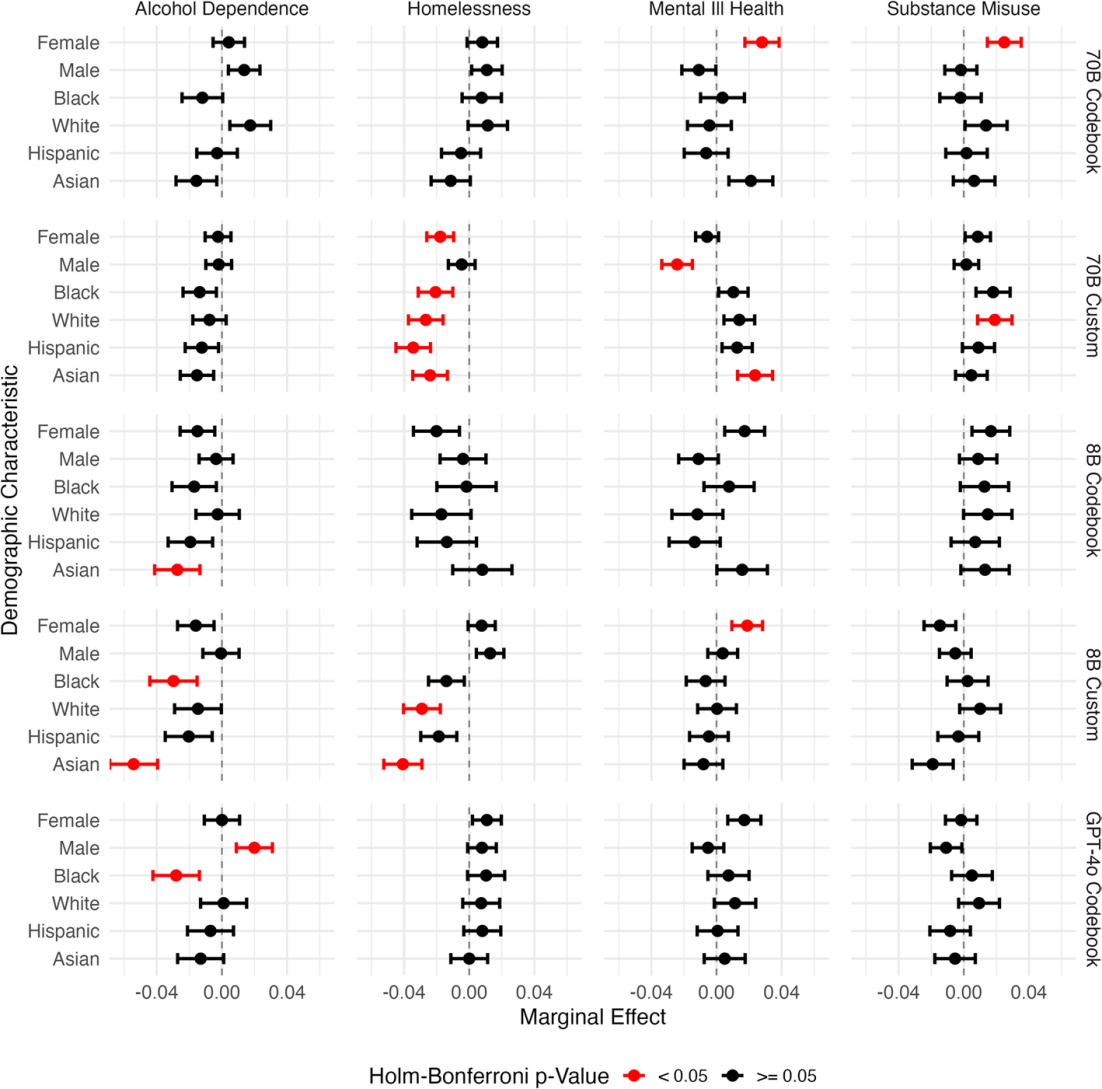
Average marginal effects of demographic characteristics (sex and race) on the probability of positive/inconclusive vulnerability classifications across different labelling configurations. Points show effect estimates relative to'unknown'baseline categories, with horizontal lines indicating 95% confidence intervals. Red points indicate effects that remain statistically significant after Holm-Bonferroni correction (p < 0.05), while black points indicate non-significant effects. Most demographic effects are small and non-significant, with few consistent patterns across vulnerabilities or labelling configurations

Figure 6 shows the AMEs and confidence intervals of the demographic features for each combination of labelling configuration and vulnerability. There are no consistent patterns across labeling configurations or vulnerability types and, after applying the Holm-Bonferroni correction for multiple comparisons, few statistically significant effects remain. The magnitudes of these effects are generally small, with the largest observed change in probability being 5.4% (for the Asian race category in the 8B custom alcohol dependence model)—this is likely negligible in the context of the variability already observed in individual label assignments.

Notable findings include a set of significant race effects for homelessness classification using the 70B custom model. All specified race categories showed a reduced likelihood of positive labelling compared to the ‘unknown’ race baseline, with effect sizes ranging from −2.07% to −3.44%. This suggests that rather than indicating differential treatment among specified races, narratives containing individuals of unspecified race are more likely to be flagged as positive for homelessness. Similar patterns, albeit with more dispersed effects, were observed for the 8B custom model in both homelessness and alcohol dependence classifications.

The largest model, GPT-4o, showed few significant demographic effects across vulnerabilities. However, in alcohol dependence classification, being black was associated with a 2.81% lower probability of a positive label and being male with a 1.99% higher probability. Unlike other models, which exhibited more varied effects across demographics, GPT-4o consistently showed little to no influence of demographic factors in most cases. This consistency makes the specific effects in alcohol dependence classification particularly noteworthy and suggests a potential area for further investigation, especially given GPT-4o’s superior overall performance.

## Discussion

Our results demonstrate that instruction-tuned large language models (IT-LLMs) can effectively support qualitative coding of police narratives, particularly in identifying and filtering cases where vulnerabilities are absent. This capability, combined with some strong performance in identifying clear positive cases with high confidence, and limited evidence of any demographic bias, suggests promising applications in augmenting traditional qualitative analysis approaches.

Our analysis reveals clear patterns in how model size and prompting strategy affect performance. While larger models demonstrated incrementally better performance with standard codebook instructions, custom prompts significantly improved the performance of smaller models with respect to their codebook counterparts. The 8-billion parameter model with custom prompts achieved F1 scores that were comparable or even superior to GPT-4o using codebook instructions (Mental Health: 0.58 vs 0.70; Homelessness: 0.75 vs 0.71; Substance Misuse: 0.67 vs 0.81; Alcohol Dependence: 0.68 vs 0.72). This finding has significant practical implications. Larger models offer clear advantages—they can effectively utilize detailed codebook instructions without extensive prompt engineering, generally achieve better performance, and may be the optimal choice when resources permit their use. However, the strong performance of smaller models with custom prompts demonstrates their viability as alternatives, particularly valuable in contexts where data security concerns preclude sharing information with proprietary LLM providers.

The models'effectiveness as negative filters emerge as one of our most promising findings. Even using simple consensus labels, all configurations demonstrated strong capabilities in identifying narratives without vulnerability indicators, with precision for negative classifications consistently exceeding 90% across vulnerabilities. This performance improves further when considering label agreement levels. At maximum agreement (10/10 classifications), the three best-performing configurations achieved remarkable precision: Custom 8B showed alignment rates of 95–99% while classifying 52–71% of examples as negative, Custom 70B achieved 98–100% alignment on 51–72% of examples, and GPT-4o reached 99–100% alignment on 49–63% of cases. These results suggest that LLMs could dramatically reduce manual coding requirements by automatically filtering out clear negative cases, allowing human coders to focus their efforts on potentially positive cases. The consistency of these results across model sizes and prompting strategies is particularly encouraging, suggesting that effective negative screening could be implemented even in contexts where smaller models are preferred for practical or security reasons.

Analysis of positive and inconclusive classifications indicates substantially lower reliability when compared with negative labels. Though performance generally improves with higher levels of agreement between repeated classifications, even the best performing configurations produce a significant number of labels that disagree with human judgement. The strongest results were observed with GPT-4o—for unanimously classified positive cases, it achieves precision exceeding 90% for both substance misuse and homelessness, though these highly confident positive classifications represent only a small portion of actual positive cases (6–11% of examples). However, model performance varies substantially across vulnerability types and becomes considerably less reliable for inconclusive classifications. Even at high agreement levels, precision for inconclusive labels rarely exceeds 70%, with considerable variation across vulnerabilities and models. This pattern aligns with confusion matrix analyses showing that models tend to err toward inconclusive labels when uncertain, frequently marking true positives as inconclusive than negative, and true negatives as inconclusive rather than positive. These findings suggest that while some positive classifications might be automated depending on one's tolerance for error, inconclusive labels appear to function more as indicators of model uncertainty than as meaningful classifications, likely warranting human review in most cases. It's worth noting that these patterns emerge in a dataset where negative cases predominate (approximately 85–90% of examples), and further research with more balanced label distributions would be valuable to fully understand the relative strengths and limitations of these models across different contexts.

The counterfactual analyses provide broad reassurance regarding demographic biases explored, while highlighting the importance of careful monitoring in new applications. After correcting for multiple comparisons, we found remarkably few statistically significant demographic effects across our configurations, and where present, their magnitudes were generally small (< 5% change in classification probability). The largest model, GPT-4o, showed particularly encouraging results, with near-zero demographic effects for most vulnerabilities. The 8B and 70B custom prompted models showed several statistically significant differences in classification rates between demographic groups, but these effects mostly suggested a tendency to label more examples as positive when demographic information isn't specified, rather than indicating differential treatment between racial groups. While these effects are minor relative to the overall classification variability we observed, they emphasize the importance of ongoing monitoring for potential biases, particularly when deploying these systems at scale where small effects could accumulate into meaningful disparities.

### Practical Implications: Benefits and Limitations

The application of IT-LLMs to qualitative coding offers several compelling advantages while raising important considerations for implementation. Perhaps most significantly, these models enable analysis at scales impractical for traditional qualitative methods. While manual coding of thousands of narratives typically requires weeks or months of sustained effort, IT-LLMs can process comparable volumes of text rapidly, enabling more comprehensive analyses of routinely collected data that have historically been constrained by resource limitations.

IT-LLM workflows also provide additional ways to maintain or enhance methodological rigor in qualitative research. The process of developing prompts for IT-LLMs inherently requires researchers to fully articulate their classification criteria through explicit codebooks and formal prompt engineering, making analytical decisions more transparent and replicable. While human coders may unconsciously supplement written definitions with implicit knowledge or reach shared understandings through discussion, IT-LLMs work solely from the explicit instructions they are given. Moreover, qualitative researchers often employ resource-intensive validation steps like intercoder reliability testing to assess coding credibility (O’Connor and Joffe 2020). While both human coders and IT-LLMs engage in complex decision-making processes that resist complete transparency, IT-LLMs'systematic nature enables rigorous investigation of their behavior patterns, biases, and limitations. Researchers can rapidly generate repeated classifications for all examples to assess consistency, vary prompt strategies to examine instruction interpretation, and systematically document where and how models succeed or fail at implementing coding criteria.

However, several important limitations warrant consideration. Despite explicit guidance to the contrary, models sometimes persist in over-interpreting behaviors as vulnerability indicators, suggesting that certain biases may be resistant to refinement through prompt engineering alone. Additionally, while IT-LLMs provide natural language explanations for their classifications, these represent post-hoc rationalizations rather than clear insights into their decision-making processes. Indeed, recent research demonstrates that LLM “self-explanations” can be misleading or unfaithful to the model's actual decision-making process (Agarwal et al 2024; Madsen et al 2024; Greenblatt et al 2024)—a phenomenon that may be analogous to human attempts to explain intuitive judgements (Nisbett & Wilson 1977). This fundamental opacity in both human and IT-LLM decision-making suggests that neither approach offers truly transparent classification processes. Rather, IT-LLMs provide different and complementary forms of analytical rigor: their systematic nature enables formal investigation of classification patterns, quantification of uncertainty in boundary cases, and documentation of specific biases in ways that may be more difficult with human coders. These characteristics make them particularly valuable for systematic large-scale classification tasks, while requiring human review to identify potential biases and misclassification patterns in each new application.

Practical implementation also raises important technical considerations. Large proprietary models like GPT-4o, while highly capable, require sharing data with third-party servers—potentially problematic for sensitive or identifiable data. Furthermore, proprietary models may be modified by developers without notice, potentially altering performance or introducing new biases. This instability necessitates ongoing validation to ensure models remain fit for purpose, particularly for longitudinal research projects. Smaller open-source models deployed locally offer greater security and the model weights can be retained and re-used for consistency, but demand more technical expertise and local computational resources.

These limitations suggest IT-LLMs are best viewed as tools to augment rather than replace traditional qualitative methods. Their ability to rapidly process large volumes of text while maintaining consistent criteria makes them valuable for initial screening and filtering tasks. However, the need for human oversight of ambiguous cases, combined with challenges around transparency and stability, indicates they should complement rather than supersede expert judgement. Used thoughtfully within these constraints, IT-LLMs offer promising capabilities for expanding the scope and of qualitative research methodology.

### Limitations and Recommendations for Future Work

#### Label Ambiguity

A fundamental challenge in this study stems from the inherent ambiguity in identifying vulnerabilities within police incident narratives. These narratives are not written with the explicit purpose of documenting specific vulnerabilities, and identifying them requires interpretation of indirect cues and contextual information. This lack of clear ground truth introduces substantial subjectivity into the labelling process, with both human and model classifications often hinging on whether described behaviors and circumstances align with expected manifestations of vulnerability. Moreover, these vulnerabilities typically exist on a continuum rather than as discrete states, making the establishment of definitive category boundaries inherently arbitrary. More generally, while ‘vulnerability’ is something of a zeitgeist in policing policy and practice, the ‘vagueness and malleability’ of the term (Brown et al. 2017) has led to variable and contested understandings.

Given this subjectivity in interpretation, our study was limited by the use of just two non-expert coders with any disagreement arbitrated by the research team, which provided only a single reference point for assessing model performance. Cases where models disagreed with human labels might represent genuine ambiguity in the narratives—instances where a larger group of human coders might show similar levels of disagreement. Additionally, domain experts, whether academic researchers or experienced police staff, may identify subtle indicators of vulnerability that both non-experts and LLMs overlook. Future work should examine how specialist knowledge affects narrative interpretation, and expand the number of coders to better understand whether apparent model errors actually reflect reasonable alternative interpretations of ambiguous cases.

#### Custom Prompt Development

Another limitation of our study involves the lack of systematic guidance on prompt engineering. While we developed custom prompts for IT-LLMs, the iterative prompt development process was conducted outside the documented experimental results, with adjustments and refinements not formally included in our reported methods. Prompt engineering is inherently iterative and interpretive, as minor changes in wording, format, or even syntax can lead to significant variations in model outputs (Chen et al 2023). However, our study does not explore how such variations affect performance, limiting the replicability of our approach for other researchers.

Further, we do not identify specific elements of our prompts that most contributed to accuracy, nor do we provide generalizable methods for adapting prompts to different datasets or coding tasks. Without a systematic evaluation of prompt variations, it is difficult to determine the extent to which our results generalize to other domains. We recommend that future work systematically examine prompt development to identify best practices, which could yield clearer guidelines for structuring prompts effectively across varied domains and tasks.

#### Practical Limitations

The application of LLMs to qualitative coding tasks presents notable practical constraints that warrant careful consideration. While these models demonstrate strong performance in replicating human coding decisions, their probabilistic nature introduces inherent variability in outputs that makes them unsuitable for case-by-case decision-making or operational deployment without human oversight. The analyses presented here demonstrate that even high-performing models produce inconsistent labels across multiple iterations, suggesting their optimal use lies in screening large volumes of data rather than making definitive classifications of individual cases.

The apparent accessibility of LLM-based methods, particularly when compared to more traditional statistical approaches, also presents both opportunities and risks. While these methods may democratize access to sophisticated text analysis capabilities, this accessibility should not overshadow the necessity for rigorous evaluation and methodological understanding. The analyses conducted in this study—including detailed assessment of human-model alignment, investigation of label variability, and examination of potential biases—represent essential components for responsible deployment.

These limitations highlight important avenues for future research regarding the broader implications of increased accessibility to sophisticated text analysis methods. It remains to be seen how non-expert analysts might implement these approaches in practice, and what decision-making processes might emerge from their widespread adoption. While traditional statistical approaches often require detailed methodological documentation and parameter specifications, LLM-based methods introduce additional complexities through prompt engineering and model selection that may not be immediately apparent to less experienced users. Future work should examine how different levels of analytical expertise influence the implementation and interpretation of these methods, and develop frameworks for ensuring their responsible deployment across varying levels of technical capability. This could include investigating standardized approaches to prompt development, establishing minimum requirements for evaluation and validation, and examining the relationship between analyst expertise and the quality of insights generated through these methods.

#### Model Advancement

As a final consideration, it is important to recognize that our evaluation represents a focused assessment of IT-LLMs on a specific task—deductive coding of vulnerability in police narratives—at a particular moment in the rapid evolution of these technologies. While our results demonstrate promising capabilities even with these early models, they likely represent a baseline rather than a ceiling for such applications. Indeed, the models'ability to follow complex instructions, reason about evidence, and rationalize their classifications suggests potential for more sophisticated applications where they act as collaborative partners throughout the qualitative coding process rather than serving purely as classification tools. Future work might explore workflows where models assist in codebook development by identifying potential ambiguities in definitions, suggest refinements based on patterns in their own uncertainty, or engage in more dynamic dialogue with research teams about challenging cases. Such applications could enhance qualitative analysis workflows in ways that thoughtfully combine human insight with machine-assisted analysis, though careful evaluation of reliability and validity would remain essential. While the present study focuses necessarily on basic classification capabilities, the models'demonstrated capacity for reasoned analysis suggests valuable directions for future research into more comprehensive applications of IT-LLMs in qualitative analysis within criminal justice contexts, and beyond.

## Data Availability

The complete dataset used in this analysis is available at https://data.boston.gov/dataset/boston-police-department-fio and is archived at https://osf.io/har9m/. All data preprocessing, label generation and analysis scripts are available in a version-controlled repository at https://github.com/samrelins/vulnerability_classifier_pipeline.These materials allow for complete reproduction of all results and figures presented in this paper.

## Appendix A: Codebook Definitions

**Code Name**: “Mental Health Difficulties”.

**General Definition**: This category should include reports of behaviors, statements, or circumstances that suggest an individual may be experiencing mental health challenges. This includes explicit evidence of mental health diagnoses, or specific behaviors related to mental health problems such as self-harm or suicidal behavior. It can also manifest as disorientation, irrational behavior, speaking incoherently, visible distress without clear cause, or descriptions of behavior that significantly deviate from social norms without an obvious immediate cause.

Categories:

1. **Positive**: Clear indications of mental health challenges based on behavior, statements, or context within the report, and having ruled out mediating factors such as intoxication or situational stressors. Examples include:
  - ⚬ Explicit discussion of mental health problems, diagnosed mental health conditions or engagement with mental health services
  - ⚬ Indications of self-harm or suicidal behavior
  - ⚬ Incoherent or nonsensical speech (in the absence of other factors such as intoxication).
  - ⚬ Disorientation or confusion about surroundings, time, or reality more generally (in the absence of other factors such as intoxication/head injury/shock).
  - ⚬ Extreme irrational behavior or reactions that are clearly unrelated to situational factors.
  - ⚬ References to hallucinations, delusions, or paranoia without any indication of drug use.
  - ⚬ Evidence of a pattern of erratic or highly unusual behavior over multiple encounters/incidents.
2. **Inconclusive**: Cases where behavior might suggest mental health difficulties, but there are possible alternative explanations, or there is insufficient evidence to determine if mental health issues are present. Examples include:
  - ⚬ Confused, erratic or delusional behavior that could be attributed to drugs/alcohol, but there isn’t evidence to confirm either way
  - ⚬ Situations where individuals exhibit extreme/disproportionate emotions or unusual behavior, where there is insufficient detail to determine if circumstances justify the behavior (e.g. extreme disagreements, seemingly unprovoked hostility, incoherent statements)
3. **Negative**: Absence of any explicit indicators of mental health issues, or indicators that can be attributed to confirmed situational factors like intoxication, anger, or typical stress responses.
  - ⚬ Rational behavior and coherent communication.
  - ⚬ Behavior clearly motivated or mediated by the circumstances e.g. intoxication or drug abuse, situational stressors, shock or head injury
  - ⚬ Unusual behavior resulting from engaging in criminal activity, interaction with the police, or attempts to conceal criminal activity from the police.

**Code Name**: “Drug Abuse”.

**General Definition**: “Drug abuse” refers to incidents where individuals are using substances known for their high potential for abuse and dependency – these may include opiates and opioids, the smoking of methamphetamine or crack cocaine, and exclude alcohol and narcotics not commonly thought to be drugs of abuse e.g. marijuana, MDMA, hallucinogens, and recreational use of cocaine. This includes behaviors or circumstances that suggest an individual is actively using these substances in a way that could be detrimental, even if negative health impacts or dependency are not detailed in the report.

Categories:

1. **Positive**: Clear indications of drug abuse based on behavior, physical evidence, or context within the report. This focuses on drugs with a high abuse potential that are typically associated with dependency and significant negative health or social outcomes. Examples include:
  - ⚬ Observable signs of impairment, that are specifically stated as relating to drug use (as distinct from alcohol or other causes). Examples include severe disorientation, drowsiness, inability to communicate coherently, profuse sweating, or physical symptoms suggesting recent high-dosage use (e.g., track marks, unconsciousness).
  - ⚬ Direct admissions of using drugs of abuse, particularly in a context suggesting habitual use or dependency.
  - ⚬ Finding an individual in possession of drugs or drug paraphernalia in a setting that strongly suggests they are actively using them. Examples include drug packaging, syringes, pipes, and spoons with residue.
  - ⚬ Emergency medical responses to overdoses of substances known for their abuse potential.
  - ⚬ Current or past involvement or engagement with drug treatment or rehabilitation organizations
2. **Inconclusive**: Cases where drug use is possible based on the context or evidence, but the information is not sufficient to conclusively determine active abuse of drugs. Examples include:
  - ⚬ Medical or police interventions where drug use is one of several possible explanations for the individual’s condition or behavior.
  - ⚬ Reports of intoxication where it is unclear if the individual is under the influence of narcotics (high) or has been drinking alcohol (drunk).
  - ⚬ Context that suggests drug use, such as the presence of drug paraphernalia, where it is unclear if any of the individuals present are actively using drugs
  - ⚬ Information detailing the use of drugs, where it is unclear if the drugs in question are drugs of abuse or are narcotics that aren’t associated with dependence (marijuana, MDMA, hallucinogens, recreational use of cocaine)
3. **Negative**: Absence of any indicators suggesting individuals are active drug abusers. The circumstances or behavior could be associated with the sale or distribution of drugs, or drinking alcohol, or the use of narcotics that aren’t considered drugs of abuse. Examples include:
  - ⚬ Incidents involving alcohol or other substances not classified as highly abusive e.g. marijuana, MDMA, hallucinogens, recreational use of cocaine
  - ⚬ Any circumstances or behavior associated with the sale or distribution of drugs or drug paraphernalia, in the absence of any evidence for active use
  - ⚬ Discussion of drug offences or convictions, where it isn’t clear that the offences relate to drug use (as opposed to supply/distribution), or that the individual is currently using drugs

**Code Name**: “Alcohol dependence”.

**General Definition**: Encompasses behaviors, statements, or circumstances that suggest an individual may be experiencing alcohol dependence or may be experiencing challenges related to excessive alcohol consumption, such as severe intoxication that notably affects their behavior during an interaction. This includes clear signs of current intoxication, references to habitual heavy drinking, and contexts that strongly imply ongoing alcohol abuse.

Categories:

1. **Positive**: Clear evidence of alcoholism or severe and problematic intoxication excluding that associated with social drinking, based on behavior, statements, or context within the report, sufficiently distinguished from other influencing factors such as drug use or transient emotional distress. Examples include:
  - ⚬ Specific evidence of frequent and heavy alcohol use (as distinct from narcotics) that suggest dependency or habitual misuse
  - ⚬ Engagement with or the recommendation of alcohol dependence services, such as AA or rehabilitation centers, where it is clear that the individual is an active drinker or is not in remission
  - ⚬ Disruptive, aggressive or problematic behavior explicitly attributed to alcohol consumption, excluding any settings associated with social drinking (bars/parties/festivals etc.)
2. **Inconclusive**: Cases where there are potential indications of alcohol-related issues, excluding those associated with social drinking, but there is a lack of sufficient information to determine if alcohol is a problem or the extent to which alcohol is a problem. Examples include:
  - ⚬ Signs of heavy intoxication, excluding social drinking, where it is unclear if the individual is under the influence of alcohol or other drugs, or has another impairment.
  - ⚬ Signs that an individual is intoxicated, excluding social drinking, but it is questionable whether the intoxication is a sign of dependence, is causing any problematic behavior, or more generally is a reason for concern.
  - ⚬ Incidents where an individual’s problematic behavior may be influenced by alcohol, excluding social drinking, but it is unclear the extent to which alcohol has contributed, or there is also significant potential for other contributing factors like mental health issues or situational stressors.
3. **Negative**: No significant indicators of excessive drinking or alcohol abuse are present, or excessive drinking in a social setting. Examples include:
  - ⚬ Any discussion of social drinking, including disruptive, problematic or aggressive behavior associated with having drunk excessively in a social setting
  - ⚬ Discussion of alcohol or evidence of drinking where there isn’t reason to suspect problem/excessive drinking

**Code Name**: “Homelessness”.

**General Definition**: Situations where individuals lack a stable, permanent, and adequate nighttime residence. This includes both explicit declarations of homelessness and implicit indicators observable through behavior, circumstances, or environmental context reported. The definition aims to identify individuals who do not have access to consistent and private nighttime accommodations, which could include those temporarily staying in shelters, cars, or other non-residential settings.

Categories:

1. **Positive**: Clear evidence that an individual is experiencing homelessness, either through self-report or unmistakable circumstances indicating a lack of stable housing. Examples include:
  - ⚬ Individuals explicitly stating they are homeless or do not have a home.
  - ⚬ Possession of a large number of personal belongings in public spaces, indicative of no permanent residence.
  - ⚬ Possession of sleeping equipment such as tents, sleeping bags or pillows in public
  - ⚬ Clear use of public spaces, squats or makeshift shelters as a primary sleeping arrangement
  - ⚬ Interactions with social or homelessness services that explicitly indicate their homeless status
2. **Inconclusive**: Cases where there are signs that may suggest homelessness, but there are possible alternative explanations, or there is insufficient evidence to definitively categories the individual as homeless. Examples include:
  - ⚬ Presence in settings commonly associated with homelessness, such as public spaces frequented by the homeless, abandoned buildings, or squats where it is unclear if the individual has stable housing
  - ⚬ Evidence of temporary or unstable residential accommodation as a primary nighttime residence e.g. hotels, AirBnBs, half-way houses, where it is suggested an individual doesn’t have a more stable alternative
  - ⚬ being found asleep in public where it is unclear if the individual may have stable housing e.g. passing out when drunk, sleeping in a car
  - ⚬ Appearing very disheveled or unkempt in a manner that suggests a lack of access to bathroom facilities
3. **Negative**: Absence of any direct evidence for homelessness. The individual’s circumstances or behavior can be clearly attributed to other factors that don’t relate to a lack of stable housing. Examples include:
  - ⚬ Individuals whose behavior is linked to other vulnerabilities such as mental health issues or intoxication, with no other signs of housing instability.
  - ⚬ loitering or causing a nuisance in public spaces without other evidence of homelessness
  - ⚬ shoplifting, begging or other criminal activity associated with, but not evidence for, homelessness

## Appendix B: Prompts

### B.1 Codebook prompt template

Read the following definition:

{{ vulnerability definition }}

You will be provided police incident reports, and should use the definition to classify the report.

Your response should begin with short notes highlighting quotes from the report, and aligning them with quotes from the definitions above. Keep your notes brief, 2 sentences max. Follow the highlighted evidence with a classification that aligns with the evidence and the definitions. Return your classification in the following format: `Classification: [POSITIVE, INCONCLUSIVE, NEGATIVE]`. Ensure that your response ends with your classification or it will be rejected.

### B.2 Parsing failure prompt

"The response you've provided does not conform to the format requested. Please classify the log in the following format:

Classification: [POSITIVE, INCONCLUSIVE, NEGATIVE]"

### B.3 Custom prompt template

You are required to classify police incident reports for involvement of persons experiencing {{ vulnerability }}. Use the following definitions for the labels you should assign:

POSITIVE: Report confirms that someone is experiencing {{ vulnerability }}, or contains unmistakable evidence of {{ vulnerability }} having ruled out any other plausible explanations. For example:

{{ positive_evidence }}

INCONCLUSIVE: Report contains evidence that is best explained by an individual experiencing {{ vulnerability }}, but there is not definitive or conclusive confirmation of {{ vulnerability }}. For example:

{{ inconclusive_evidence }}

NEGATIVE: Evidence for {{ vulnerability }} that can be explained by other factors, or no evidence for {{ vulnerability }}. The following should not be considered evidence for {{ vulnerability }}:

{{ negative_evidence }}

Write short notes highlighting quotes from the report, and link each quote to the relevant quote above. Keep the notes to two sentences max.

End your report with a classification that aligns with the evidence you have highlighted. Use the format “Classification: [POSITIVE, INCONCLUSIVE, NEGATIVE]”. If the final word of your report is not the classification, it will be marked invalid.

### B.4 Mental ill health evidence

Positive Evidence:

- Statements that someone is experiencing mental health difficulties, has or is receiving treatment for a mental health diagnosis
- Individuals engaging with or being offered mental health services
- Clear and obvious descriptions of emotional disturbance, irrational or erratic behavior, statements or actions suggesting delusions or loss of contact with reality, where there is no other reasonable explanation other than someone experiencing mental health issues
- Deliberate self harm or attempts at suicide

Inconclusive Evidence:

- –Clear and obvious symptoms or behavior that are best explained as mental health related, but there are other possible explanations for the behavior symptoms (e.g. intoxication, circumstances not detailed in the report)

Negative Evidence:

- any evidence associated with drug/alcohol use or dependence, overdose or detox/rehab services
- any evidence associated with homelessness or street outreach activity
- general stress, anger, aggression, hostility, panic or anxiety
- strange or unusual behavior related to criminal activity or attempts to conceal criminal activity
- Anxiety in the presence of the police, or strange, erratic or aggressive behavior towards the police
- Evidence of medical treatment or medicines that are not confirmed to be mental health related

### B.5 Substance Misuse Evidence

Positive Evidence:

- Explicit statements that someone is currently using drugs or is under the influence of drugs
- Individuals engaging with or being offered drug rehabilitation services or organizations

Inconclusive Evidence:

- possession or presence of drugs or drug paraphernalia, with strong evidence that the individuals present are actively using drugs
- Discussion of rehabilitation services for an active addiction (excluding general street outreach services), that might be drug or alcohol related but it isn't specified
- Statements that an individual is intoxicated where it is unclear if the intoxicant is alcohol or other narcotics

Negative Evidence:

- Any drug related activity, possession, sale or trafficking that isn't accompanied by specific evidence that an individual is using drugs or has a drug abuse problem
- Marijuana use or possession
- Any evidence associated with alcohol, homelessness, or mental health conditions

### B.6 Alcohol Dependence Evidence

Positive Evidence:

- Explicit statements that someone is an alcoholic, frequently drinks heavily, or is dependent on alcohol
- Discussion of alcoholism services, such as rehabilitation centers (specifically for drinking) or AA
- Disruptive, aggressive or problematic behavior that is explicitly attributed to alcohol consumption, excluding settings associated with social drinking (bars/parties/festivals etc.)

Inconclusive Evidence:

- Disruptive, aggressive or problematic behavior that is clearly attributed to intoxication, but it is not clear if drugs or drugs or alcohol are the intoxicant
- Confirmation of extreme alcohol intoxication, where it isn't clear if it is a recurrent problem
- Instances where extremely problematic behavior co-occurs with alcohol consumption, but the extent of the influence of alcohol is unclear

Negative Evidence:

- Disruptive, aggressive or problematic behavior that isn't clearly attributed to intoxication
- Evidence of drinking, including intoxication in social drinking settings—bars/festivals/parties
- The presence of alcohol or alcohol containers where there isn't a clear reason to suspect problem drinking
- Any evidence relating to drug abuse, overdose, or homelessness

### B.7 Homelessness Evidence

Positive Evidence:

- Statements that someone is homeless or does not have any nighttime accommodation
- Individuals engaging with or being offered homelessness services or organizations
- Individuals being found with makeshift sleeping/living arrangements on the street or in unstable living environments

Inconclusive Evidence:

- Strong evidence that someone does not have any nighttime accommodation, but is not definitive
- Being found asleep in public

Negative Evidence:

- Causing a nuisance, loitering, or being trespassed from places where homeless individuals may congregate
- Use of detox or rehab services for alcohol or drug abuse
- Any evidence or behavior associated with a person's drug use, alcoholism, mental health difficulties, or sex work
- Any vague or uncooperative responses to police questioning about address information that don't result in an admission of homelessness

## Appendix C: Label Distributions in Model Outputs

Label distributions generated by different LLM configurations during our initial coding of 4,000 narratives.

Tables 1 and 2

**Table 1:** Consensus Label Proportions

Vulnerability	Label	Custom 8B	Custom 70B	Codebook 8B	Codebook 70B
**Alcohol Dependence**	**Negative**	94.2	96.15	45.975	89.95
	**Inconclusive**	5.275	3.475	27.5	8.325
	**Positive**	0.525	0.375	26.525	1.725
**Substance Misuse**	**Negative**	87.9	87.35	42.925	74.7
	**Inconclusive**	9.225	8.625	30.675	17.125
	**Positive**	2.875	4.025	26.4	8.175
**Homelessness**	**Negative**	93.725	88.925	51.75	74.05
	**Inconclusive**	3.275	7.35	36.725	21.925
	**Positive**	3.0	3.725	11.525	4.025
**Mental Ill Health**	**Negative**	93.2	95.8	47.5	81.725
	**Inconclusive**	5.225	2.875	26.425	14.175
	**Positive**	1.575	1.325	26.075	4.1

Distribution of consensus labels across different labelling configurations and vulnerabilities. Values show the percentage of narratives assigned each label type (negative, inconclusive, positive) based on majority voting across 10 iterations. Results demonstrate that custom prompts generally produced more negative classifications than codebook prompts, with the effect particularly pronounced for smaller models.

**Table 2:** Unanimous Negative Label Proportions

Vulnerability	Codebook 70B	Codebook 8B	Custom 70B	Custom 8B
Alcohol Dependence	84.15	12.0	94.15	90.525
Substance Misuse	65.05	13.05	82.775	80.75
Homelessness	57.15	14.225	83.25	90.775
Mental Ill Health	70.4	15.0	92.825	88.1

Proportion of narratives receiving unanimous negative labels (10/10 votes) across different labelling configurations and vulnerabilities. Results show that custom prompts achieved consistently higher rates of unanimous negative classifications compared to codebook prompts, with larger models generally producing more unanimous classifications than smaller ones.

## Footnotes

1 Full text of the PDDL can be found at: http://opendatacommons.org/licenses/pddl/1.0/

2 Links to all program code required to replicate our analyses, including pre-processing steps, can be found in Sect. 2.7 Reproducibility Materials.

3 For a recent review of Large Language Models, their underlying technologies, characteristics and use cases interested readers are directed to Naveed et al. (2023).

4 The quantity of narratives and choice of Llama models for initial coding were solely determined by the cost implications of using cloud-computing services from which models were accessed.

5 Again, the selection of 500 narratives was simply constrained by resources available.
